# Comparative Survey of Entomophagy and Entomotherapeutic Practices in Six Tribes of Eastern Arunachal Pradesh (India)

**DOI:** 10.1186/1746-4269-9-50

**Published:** 2013-07-19

**Authors:** Jharna Chakravorty, Sampat Ghosh, V Benno Meyer-Rochow

**Affiliations:** 1 Biochemical Nutrition Laboratory, Department of Zoology, Rajiv Gandhi University, Arunachal Pradesh 791112, India; 2 School of Engineering and Science, Jacobs University, Research II (Rm. 37), D-28759 Bremen, Germany; 3 Department of Biology, Oulu University, Linnanmaa Campus, P.O. Box 3000, FIN-90014 Oulu, Finland

**Keywords:** Edible insects, Wangcho (Wancho), Nocte, Singpho, Tangsa, Deori, Chakma, Traditional wisdom, Biodiversity

## Abstract

A consolidated list of edible insects used in the eastern part of Arunachal Pradesh (N.E. India) by Wangcho (Wancho) and Nocte tribes of the Tirap District and the Shingpo, Tangsa, Deori and Chakma of the Changlang District has been prepared. The list is based on thorough, semi-structured field-interviews with 20 informants of each tribal group. At least 51 insect species, belonging to 9 orders were considered edible. The largest number of the edible species belonged to the Coleoptera (14), followed by 10 each of the Orthoptera and Hymenoptera, 9 of the Hemiptera, 3 Lepidoptera, 2 Isoptera and one each of Ephemeroptera, Odonata and Mantodea. As far as therapeutic uses of insects are concerned, 4 species (Hemiptera) were mentioned by the Wangcho (Wancho). Food insects are chosen by members of the various tribes according to traditional beliefs, taste, regional and seasonal availability of the insects. Depending on the species, only certain, but sometimes all, developmental stages are consumed. Preparation of the food insects for consumption involves mainly roasting or boiling. With the degradation of natural resources, habitat loss, rapid population growth, and increasing ‘westernization’ , the traditional wisdom of North-East Indian tribals related to insect uses is at risk of being lost.

## Introduction

Insects are a genuine food item for humans in numerous countries of the world, but as the recent global survey by Mistuhashi [1] has shown, information specifically on India in this regard is patchy and scant. Our review [2] confirmed this and to remedy this sorry state of affairs, we singled out the North-East of India with its multitude of indigenous tribes as the area for our ethno-entomological field work. Our long-term goal is to obtain complete lists of all edible insects for all tribes of the region and to record folk medicinal uses of insects and other arthropods.

Our earlier resesrch has dealt with aspects of entomophagy (i.e., the consumption of insects by humans) amongst tribal members of the Ao-Naga [3,4], Meeteis [4], Khasi [4], Nyishi [5] and Galo [5]. In this paper we expand the study to include observations on tribals of the districts of Tirap and Changlang of Eastern Arunachal Pradesh. The region is remote and difficult to reach and the tribals surveyed (i.e., Nocte, Wangcho (Wancho), Tangsa, Singpho, Deori, and Chakma) have their own languages and traditions.

The ethno-entomological knowledge of the local inhabitants is extensive and handed down orally from generation to generation, but people outside the communities in question are rarely aware of this store of knowledge. Still available, it is, however, more and more in danger of being lost. Problems abound, for much of the region in question is not only hard to reach, but special permits to enter the tribal regions are required from the Indian administration. Moreover, local informants are reluctant to have their voices recorded and rather remain silent when they notice that what they reveal is being taped. There is, thus, always the risk that the information obtained from local informants (tribals of the remote regions often neither speak Hindi nor English so that an interpreter is needed) is to some extent incomplete. Yet, this difficulty needs to be seen as a minor set-back in the context of the massive threats that the traditional uses of insects face in these societies.

These major threats are principally of three kinds. Given that collecting and using insects are still regular components of the local ways of life, an increasing population is likely to require more and more insects. Thus, pressure, especially on the most sought-after species, could reach an extent that is unsustainable. Moreover, the loss of certain species or significant reductions in the number of individuals of ecologically important species due to overuse (for example large numbers of dragon flies collected as nymphs and the subsequent absence of predatory adults) could change the ecological balance in unpredictable ways.

The second threat comes from deforestation and climate change and in both cases could best be described as habitat loss. In the wake of such changes that are likely to affect the stability of the environment, important species of insects (and with them the uses they used to be put to) could disappear. Undoubtedly, there would be other ecological consequences.

The third threat comes from an increase in the introduction and acceptance of western food items and western medicines, both of which eroding and ultimately replacing the traditional uses of insects as food and as components of local therapies administered to treat a variety of health conditions.

For these reasons, we see it as an imperative and urgent task to document as much as we can of the traditional wisdom as long as it is still possible.

## Materials and methods

**Figure 1:**
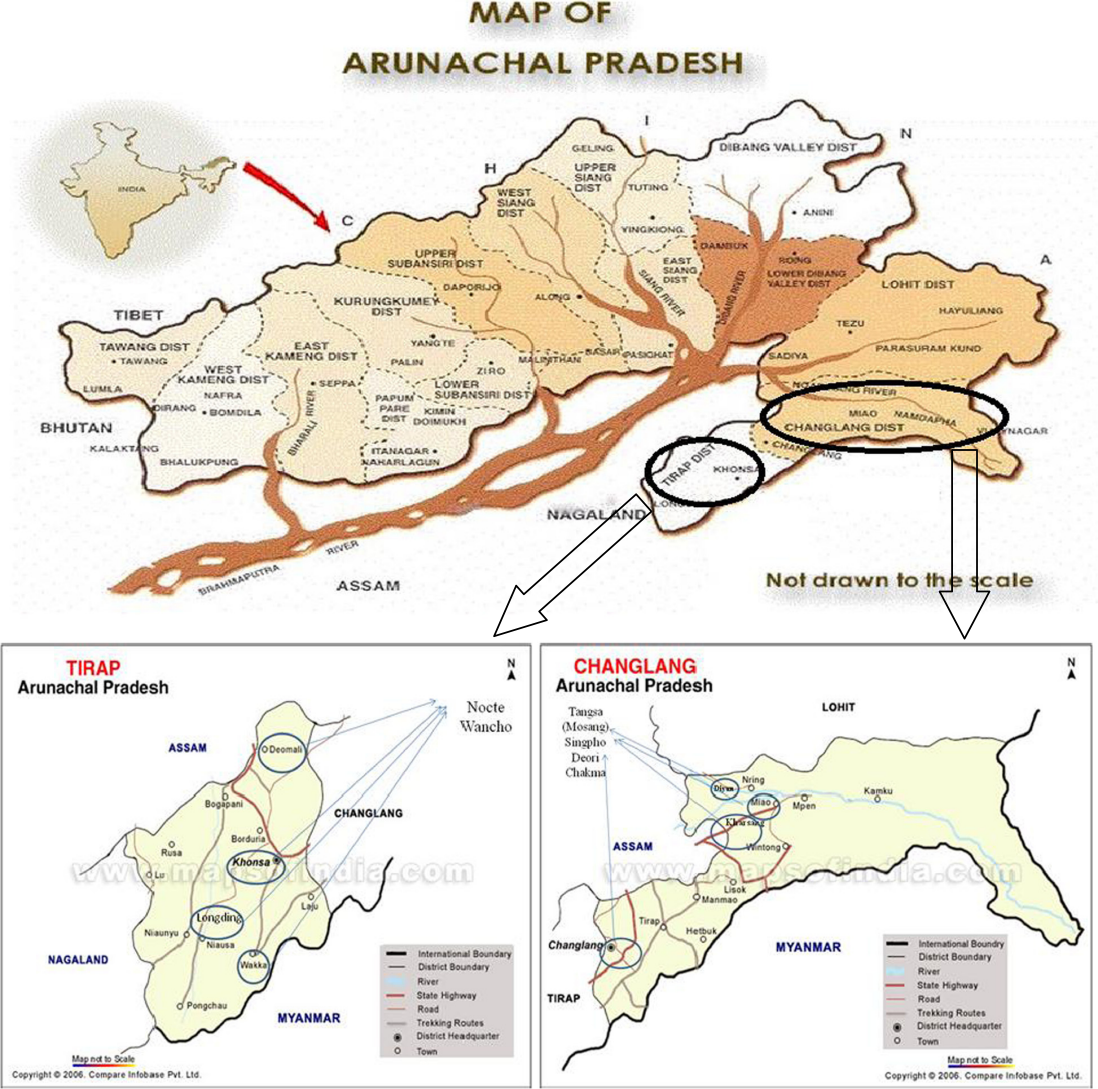
**Map of Arunachal Pradesh indicating the study area.** Map of Tirap district of Arunachal Pradesh showing study site (Map adopted from http://www.mapsofindia.com). Map of Changlang district of Arunachal Pradesh showing study site (Map adopted from http://www.mapsofindia.com).

Extensive field surveys to record the various uses of insects amongst members of the tribes of Eastern Arunachal Pradesh namely Nocte, Wangcho (Wancho), Singpho, Tangsa, Deori and Chakma were carried out during the months of March (2 weeks) and April/May (10 days) in the two districts of Tirap and Changlang in the north-east Indian state of Arunachal Pradesh (Figure 1). Ten villages, selected at random, were visited in each of these two tribal areas. The number of households per village was 20–25. At least two to three households inhabited by village elders and their families were visited. Recommendations by the headman or village elders to interview certain knowledgeable persons in another village were sometimes followed. The surveys were based on interviews during which a total of 20 persons aged between 45 and 70 years of age (12 male and 8 female) from each tribe were shown museum specimens or photographs of insects.

Kind persistence and expressing a positive attitude towards the consumption of insects by humans were necessary to create an environment of trust. The interviewed people were then asked, often with the help of a local interpreter, simple questions in order to obtain information on the vernacular names of the edible or otherwise important insects, on seasonal availabilities, stages of insects consumed or used, mode of preparation, assumed therapeutic value, folklore related to insects and anything else deemed important in connection with the insect in question. As the knowledge of Hindi or English of the locals was often not great or nonexistent, our questions had to be simple and to the point. Some insects available at the times of the survey were collected by locals from different habitats, e.g., soils and farmland, shrubs and trees, grassland and dwellings. They were then preserved according to standard methods [6] and identified with the help of published keys [7-10]. Where this was not possible, the insects were sent to Kolkata to be identified by entomological experts of the Zoological Survey of India.

According to the website of the district of Tirap (http://www.tirap.nic.in), maintained by the National Information Center, the region in question lies between latitudes 26°38’N to 27°47’N and longitudes 96°16’E to 95°40’E. It is bounded by Myanmar towards the south; the Changlang district towards the east; the Dibrugarh district of Assam in the north and Sibsagar (Assam) and Mon districts (Nagaland) towards the west. Tirap covers a total area of 2362 sq.km and has a population of 100,227 (2001 census), of which 52,461 are males and 47,766 females. The population density of 42 inhabitants per sq. km is the highest in the state of Arunachal Pradesh (approx. 13 per sq. km). The average literacy rate of the inhabitants of Tirap is 42.01% and in the decade between 1991 and 2001, the population of Tirap grew by 14,759 (corresponding to a 17.21% increase).

The principle tribes (i.e., 70% of the total population) that inhabit the district of Tirap are the Wangcho (Wancho), Nocte, and Tutsa. Of these tribal groups, the Wangcho (Wancho) make up about 50%, Nocte approximately 45%, and Tutsa 5%. Each of the three tribes occupies a distinct geographical area and has its own social norms, customs, beliefs and practices. Although warfare used to be common between them and all are martial Naga tribes, they now live in social harmony with each other, clinging to their own cultures. Their life-style is community based and their livelihood depends on farming and other activities such as contract work in forests, trade in local products, agricultural labour, government employment, etc. The agriculture is primarily of the shifting type (jhum), although people have started to adopt terrace farming as well.

The altitudes vary from around 60 m above sea level in the north-west to about 1,500 m in mountainous areas. The climate varies considerably from place to place due to the altitude and nature of the terrain: it is generally cool and highly humid at the elevations and in the valleys. The cold season prevails from the latter part of November to late February and is followed by pre-monsoon weather from March to May, while the actual monsoon season lasts from May to about mid October.

According to the website of the Changlang District (http://www.changlang.nic.in), maintained by the National Information Center, Singpho, Tangsa, Deori and Chakma are the principle inhabitants of the Changlang district, which lies between the latitudes 26°40’N and 27°40’N, and longitudes 95°11’E and 97°11’E. It is bounded by the districts of Tinsukia (Assam) and (Arunachal Pradesh) in the north, by Tirap in the west and by Myanmar in the south-east. Chanlang has an area of 4662 sq. km and a population of 125,334 (2001 Census), of which as per 2001 census 65,759 are males and 59,575 are females. The literacy rate is 51.98 percent and the decennial growth rate of the population during 1991–2001 was 30.84 for the district. The aboriginal inhabitants of the Changlang district are the Tangsa, Singpho and Tutsa. The Tangsa tribe comprises a number of sub-tribes, namely the Muklom, Havi, Longchang, Mossang, Jugli, Kimsing, Ronrang, Mungrey, Longphi, Longri, Ponthai, Sangwal, Tikhak, Yungkuk, Sakieng and Thamphang. All of these occupy the south-eastern hills of the district along the Indo-Myanmar border and the Namchik Basin and differ slightly from each other in dialect and customs.

The Singpho inhabit the area of the plains and foothills of the northern part of the district while the Tutsa live in the western part of the district. Other tribes who have migrated to the district are Nocte, Lisu (Yobin), and Deori. The Chakma came as refugees from Bangladesh. The climatic conditions in this District vary from place to place due to the mountainous nature of the terrain; its average altitude is 580 m.

Places like Miao, Kharsang, Jairampur, Bordumsa and Diyun, which are located at lower elevations and in the valleys, experience hot and humid weather in summer (June-August), but in the hill areas the climate is moderate and pleasant. Between December and February it is cold. January is the coldest month when average maximum and minimum temperatures are about 22°C and 13°C, respectively. August is the hottest month, during which temperatures may occasionally exceed 30°C. Annual average maximum and minimum temperatures are 26.96°C and 18.63°C. Like temperature, rainfall is also much influenced by the terrain, ranging from 3800 mm to 4866 mm per annum. Major rainfall is received during June through October.

## Results and discussion

**Figure 2:**
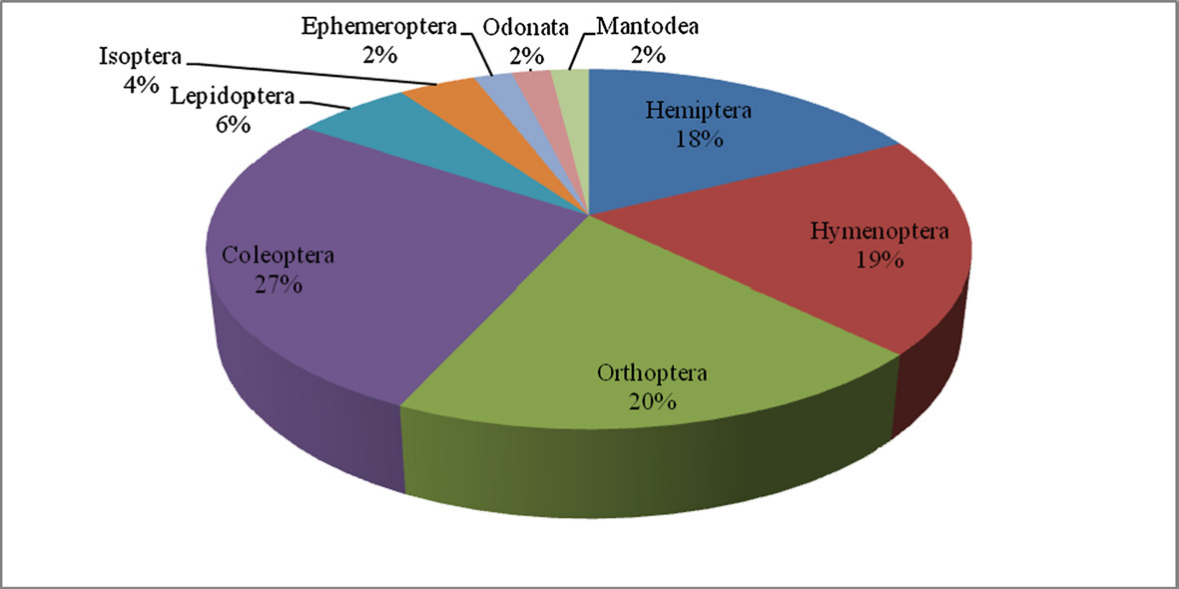
Orderwise distribution of edible insects used by Eastern Arunachal tribes.

The present study revealed that a total of 51 insect species (including both identified and unidentified species), principally belonging to 21 families and 9 orders, find acceptance as food by the local ethnic people. Species distribution is such that Ephemeroptera, Odonata and Mantodea were represented by one species each; 10 species each belonged to the orders Orthoptera and Hymenoptera, 9 were representatives of the Hemiptera (including Homoptera), 14 of the Coleoptera, 3 of the Lepidoptera, and 2 of the Isoptera. Figure 2 shows the order-wise distribution of the identified edible insects of the tribes studied. However, the list of edible insects is likely to be incomplete and probably much longer, because we cannot rule out the possibility of additional species accepted as food, but not present or not shown to the local people at the time of our interviews. Some information might also have deliberately been withheld for reasons of taboos associated with certain species or a feeling of shame to admit their consumption [11].

An inventory of the identified edible insects of the six ethnic tribes of the studied area is presented in Tables 1, 2, 3, 4, 5, 6. The Wangcho (Wancho) were found to consume at least 20 insect species, including some with therapeutic uses. Of these 20 species, 4 were Orthoptera, 6 were Hymenoptera, 7 were Hemiptera, and 3 were Coleoptera (Table 1). Out of the 7 Hemiptera, 4 were reported by Wangcho (Wancho) informants to be exclusively used for therapeutic purposes. Except for the Wangcho (Wancho), no members of the other tribes mentioned any therapeutic uses of insects, but this does not mean that they had absolutely no medicinal uses for them. More likely only a few traditional healers would know (or be allowed) to disclose such uses. The use of honey as a remedy for a variety of ills, however, was widely known to the members of all tribes.

**Table 1:** Inventory of edible insects used by members of the Wangcho (Wancho) tribe of Eastern Arunachal Pradesh

**Scientific name**	**Order**	**Family**	**English name**	**Vernacular name**	**Seasonal availability**	**Mode of intake**	**Remarks**
*Chondacris rosea* (DeGeer 1773)	Orthoptera	Acrididae	Short horn grasshopper	Okuk	Sept- Nov	Head, appendages and wings are discarded and bodies are roasted	
Unidentified	Orthoptera	Acrididae	Short horn grasshopper	Okuk	Sept- Oct	roasted	
*Brachytrupes* sp.	Orthoptera	Gryllidae	Cricket	Okuk	May- Sept	Head, appendages and wings are discarded, bodies are roasted	
*Brachytrupes orientalis* (Burmeister 1838)	Orthoptera	Gryllidae	Cricket	Okuk	May- Sept	Head, appendages and wings are discarded, bodies are roasted	
*Aspongopus nepalensis* Westwood 1837	Hemiptera	Pentatomidae	Bug	Longvia	Dec- Feb	Raw paste with chili and ginger	Sometimes consumption causes psychiatric disorder
*Halyomorpha picus* (Fabricius 1794)	Hemiptera	Pentatomidae	Bug	Nyajong waekhoi	Nov- Feb	Suck the sting in cold and cough	Not reported as a normal food item for Wangcho (Wancho) people, but used for therapeutic purposes
*Nezara viridula* (Linnaeus 1758)	Hemiptera	Pentatomidae	Bug	Jakwikhoi	Dec- Feb	Wings are discarded, roasted	--
*Rhynchoris humeralis* (Thunberg 1783)	Hemiptera	Pentatomidae	Bug	Viapath	Jan- Mar	People use to press the lower portion of the insect to remove the stink gland, wings are discarded and insect is roasted	The secretion is poisonous for the eye, yellowish in colour and it takes one week to remove it from the hand
*Dalader acuticosta* (Amyot & Serville 1843)	Hemiptera	Pyrrhocoridae	Bug	Waekhoi	Dec- Feb	Suck the sting in cold and cough	Not reported as a normal food item for Wangcho (Wancho) people, but used for therapeutic purposes
*Mictis tenebrosa* (Fabricius 1787)	Hemiptera	Pyrrhocoridae	Bug	Waekhoi	April- Aug	Suck the sting in cold and cough	Not reported as a normal food item for Wangcho (Wancho) people, but used for therapeutic purposes
*Antilochus coqueberti* (Fabricius 1803)	Hemiptera	Pyrrhocoridae	Bug	Wanwikhoi/ Wanchong	Around the year	Raw or roasted	Species is used only for therapeutic purposes
*Dorcus* sp.	Coleoptera	Lucanidae	Stag beetle	Mogap magai	Aug- Oct	Appendages discarded, roasted	
Unidentified	Coleoptera	Lucanidae	Stag beetle	Mogap magai	Jul- Sept	Appendages discarded, roasted	
Unidentified small species	Coleoptera			Mophong molong/ Notphong	June - Sept	Boiled or roasted	
*Apis mellifera* Linnaeus 1758	Hymenoptera	Apidae	Honeybee	Nakat	Nov- Jan	Honey and sometimes larvae, pupae and queen boiled	
*Apis* sp.	Hymenoptera	Apidae	Honeybee	Nakat	Nov –Jan	Honey and sometimes larvae, pupae and queen boiled	
*Xylocopa sp*	Hymenoptera	Xylocopidae	Bee	Nakat	Nov- Mar		
Unidentified	Hymenoptera	Vespidae	Wasp	Nahtam	Nov- Jan	Immature stages raw or roasted nymph and queen boiled	
Unidentified	Hymenoptera	Vespidae	Wasp	Thokananu	Oct- Dec	Larvae	
*Oecophylla smaragdina* Fabricius 1775	Hymenoptera	Formicidae	Red ant	Thajao	Round the year	Pupae are consumed	

**Table 2:** Inventory of edible insects used by members of the Nocte tribe of Eastern Arunachal Pradesh

**Scientific name**	**Order**	**Family**	**English name**	**Vernacular name**	**Seasonal availability**	**Mode of intake**	**Remarks**
Unidentified	Ephemeroptera		Mayfly	Along/ Phin	Sept- Nov	Roasted	
*Termes* sp.	Isoptera	Termitidae	Termite	Akhun	May- June	Roasted with local edible leaves, wings discarded	
*Odontotermes* sp.	Isoptera	Odontotermitidae		Akhun	May- June	Roasted with local edible leaves, wings discarded	
Unidentified	Odonata	Gomphidae	Odonate larvae of dragonfly	Moogi	Sept - Oct	Roasted with local edible leaves	
*Brachytrupes* sp.	Orthoptera	Gryllidae	Cricket	Loothong/ Idmon/ Churu	May- Sept	Antennae, wings and anal cerci discarded, bind with local leaves and roasted	
*Brachytrupes orientalis* (Burmeister 1838)	Orthoptera	Gryllidae	Cricket	Loothong/ Idmon/ Churu	May- Sept	Antennae, wings and anal cerci discarded, bind with local leaves and roasted	
*Macrolyristes* sp.	Orthoptera	Tettigoniidae	Katydid	Kokchug/ Headboon	Sept- Oct	Antennae, wings and anal cerci discarded, bind with local edible leaves and roasted	
Unidentified	Orthoptera	Tettigoniidae	Katydid	Kotkot	Aug- Nov	Roasted	
*Aspongopus nepalensis* Westwood 1837	Hemiptera	Pentatomidae	Bug	Longheto/ Seve	Dec- Feb	Boiled or prepared as chutney	Sometimes consumption causes psychiatric disorder
*Tessaratoma quadrata* Distant 1902	Hemiptera	Pentatomidae	Bug	Siviliangkhan/ Heta	Jan- Mar	Wings and appendages discarded, bodies are roasted (“gi”)	Nocte people reported secretion from backside is quite dangerous for eye
*Halyomorpha picus* (Fabricius 1794)	Hemiptera	Pentatomidae	Bug	Longliasivi/ Longlia	Nov- Feb	Wings and appendages discarded, bodies are roasted (“gi”)	
*Apis (cerana) indica* (Fabricius 1798)	Hymenoptera	Apidae	Honeybee	Nyakui	Nov- Jan	Immature stages are consumed, boiled or roasted	Honey is used widely as medicinal agent
*Apis mellifera* Linnaeus 1758	Hymenoptera	Apidae	Honeybee	Nyakui	Nov- Jan	Immature stages are consumed, boiled or roasted	Honey is used widely as medicinal agent
Unidentified	Hymenoptera	Apidae or Vespidae	Bee or wasp	Nyakui	Nov - Jan	Immature stages are consumed, boiled or roasted	
*Xylocopa sp*	Hymenoptera	Xylocopidae	Bee	Nyakui	Nov- Mar	Immature stages are consumed, boiled or roasted	
*Oecophylla smaragdina* Fabricius 1775	Hymenoptera	Formicidae	Red ant	Aukhithio/Thapi/Khawa	Round the year	People use to take the hive and treat it with hot water, keep it as it is in pan and roasted it with masala	

**Table 3:** Inventory of edible insects used by members of the Singpho tribe of Eastern Arunachal Pradesh

**Scientific name**	**Order**	**Family**	**English name**	**Vernacular name**	**Seasonal availability**	**Mode of intake**	**Remarks**
*Termes* sp.	Isoptera	Termitidae	Termite	khukan	May- June	Wing are discarded, fried	
*Odontotermes* sp.	Isoptera	Odontotermitidae		khukan	May - June	Wings are discarded, fried	
*Chondacris rosea* (DeGeer 1773)	Orthoptera	Acrididae	Short horned grasshopper	Macherie	Sept- Nov	Head, appendages and wings are bodies are discarded and roast	
*Brachytrupes* sp.	Orthoptera	Gryllidae	Cricket	Gdun	May- Sept	Head, antennae and legs are discarded and bodies are roasted	
*Brachytrupes orientalis* (Burmeister 1838)	Orthoptera	Gryllidae	Cricket	Gdun	May- Sept	Head, antennae and legs are discarded and bodies are roasted	
*Aspongopus nepalensis* Westwood 1837	Hemiptera	Pentatomidae	Bug	Chammah	Dec- Feb	Fried or roasted used after removal of stink gland that tastes bitter	Sometimes consumption causes psychiatric disorder
*Mictis tenebrosa* (Fabricius 1787)	Hemiptera	Pyrrhocoridae	Bug	Chammah	April- Aug	Boiled with vegetables	Singpho people reported that these insects are collected from rotten bamboo
Unidentified	Hemiptera	Cicadidae	Cicada	Machera	April- Aug	Roasted	
*Xylotrupes gideon* Guérin-Méneville 1830	Coleoptera	Scarabaeidae	Beetle	Chingiet	May- July	Legs discarded, roasted or fried	
*Batocera roylei* Hope 1833	Coleoptera	Cerambycidae	Long horn beetle	Chingiet	June- Aug	Antennae and wings discarded, roasted	
*Apis (cerana) indica* (Fabricius 1798)	Hymenoptera	Apidae	Honeybee	Lagat	Nov- Jan	Immature stages and honey is being consumed	Honey is used widely as medicinal agent
*Apis mellifera* Linnaeus 1758	Hymenoptera	Apidae	Honeybee	Lagat	Nov- Jan	Immature stages and honey are being consumed	Honey is used widely as medicinal agent
*Eumenes* sp.	Hymenoptera	Vespidae	Wasp	Katpatkai	Nov- Dec	Generally larval stages are being consumed, when wings are not developed, fried	
*Oecophylla smaragdina* Fabricius 1775	Hymenoptera	Formicidae	Red ant	Makhao	Round the year	Pupae boiled or fried	

**Table 4:** Inventory of edible insects used by members of the Tangsa tribe of Eastern Arunachal Pradesh

**Scientific name**	**Order**	**Family**	**English name**	**Vernacular name**	**Seasonal availability**	**Mode of intake**	**Remarks**
*Aspongopus nepalensis* Westwood 1837	Hemiptera	Pentatomidae	Bug	Shiphon	Dec- Feb	Fried or roasted after removal of stink gland that tastes bitter	Sometimes consumption causes psychiatric disorder
*Tessaratoma quadrata* Distant 1902	Hemiptera	Pentatomidae	Bug	Shipho/ Nagoo	Jan- Mar	Roasted	
Unidentified	Hemiptera	Cicadidae	Cicada	Kapchera	April- Aug	Roasted	Generally consumed by children
*Apis (cerana) indica* (Fabricius 1798)	Hymenoptera	Apidae	Honeybee	Yankung/ yakay	Nov- Jan	Immature stages and honey is being consumed; larvae are also being consumed, boiled	
*Apis mellifera* Linnaeus 1758	Hymenoptera	Apidae	Honeybee	Yakay	Nov- Jan	Immature sages and honey are being consumed	Honey is used widely as medicinal agent
Unidentified	Hymenoptera	Vespidae	Wasp	Yanjung	Oct- Dec	Immature are being consumed	
*Eumenes* sp.	Hymenoptera	Vespidae	Wasp	Longli	Nov- Dec	Generally larval stage is being consumed, when wings are not developed, fried	
*Vespa orientalis* Linnaeus 1771	Hymenoptera	Vespidae	Hornet	Yandok	Nov- Feb	Immature stages are boiled	
*Oecophylla smaragdina* Fabricius 1775	Hymenoptera	Formicidae	Red ant	Saisho	Round the year	Larvae and pupae boiled or fried	

**Table 5:** Inventory of edible insects used by members of the Deori tribe of Eastern Arunachal Pradesh

**Scientific name**	**Order**	**Family**	**English name**	**Vernacular name**	**Seasonal availability**	**Mode of intake**	**Remarks**
*Chondacris rosea* (DeGeer 1773)	Orthoptera	Acrididae	Short horn grasshopper	Phoring	Sept- Nov	Legs and wings are discarded, roasted or fried with mustard oil	
*Laptysma* sp.	Orthoptera	Acrididae	Short horn grasshopper	Phoring	Aug- Oct	Legs and wings discarded, roasted or fried with mustard oil	
Unidentified	Orthoptera	Acrididae	Short horn grasshopper	Phoring	Sept- Oct	Legs and wings are discarded, roasted or fried with mustard oil	
*Brachytrupes* sp.	Orthoptera	Gryllidae	Cricket	Shingapok	May- Sept	Wings, legs and antennae discarded and bodies are fried with mustard oil	
*Brachytrupes orientalis* (Burmeister 1838)	Orthoptera	Gryllidae	Cricket	Shingapok	May- Sept	Wings, legs and antennae discarded and bodies are fried with oil	
Unidentified	Orthoptera	Tettigoniidae	Katydid	Phoring	Sept- Oct	Legs, antennae and wings are discarded, roasted or fried with oil	
*Apis mellifera* Linnaeus 1758	Hymenoptera	Apidae	Honeybee	Moumakhi	Nov- Jan	Honey	
*Apis* sp.	Hymenoptera	Apidae	Honeybee	Moumakhi	Nov- Jan	Honey	
*Oecophylla smaragdina* Fabricius 1775	Hymenoptera	Formicidae	Red ant	Semete	All year round	Pupae	
*Samia ricini* (Donovan 1798)	Lepidoptera	Saturniidae	Saturniid silk moth	Palu	April- Sept	Large caterpillar stages, pupae boiled and fried	
*Antheraea assamensis* Helfer 1837	Lepidoptera	Saturniidae	Muga silk moth	Palu	May- Sept	Large caterpillar stages, pupae boiled and fried	
*Bombyx mori* (Linnaeus 1758)	Lepidoptera	Bombycidae	Silkworm or mulberry silk worm	Palu	May- Sept	Large caterpillar stages, pupae boiled and fried	

**Table 6:** Inventory of edible insects used by members of the Chakma tribe of Eastern Arunachal Pradesh

**Scientific name**	**Order**	**Family**	**English name**	**Vernacular name**	**Seasonal availability**	**Mode of intake**	**Remarks**
*Chondacris rosea* (DeGeer 1773)	Orthoptera	Acrididae	Short horn grasshopper	Phiring	Sept- Nov	Roasted or fried with mustard oil	
*Brachytrupes* sp.	Orthoptera	Gryllidae	Cricket	Gumro	May- Sept	Wings discarded and fried with mustard oil	
*Brachytrupes orientalis* (Burmeister 1838)	Orthoptera	Gryllidae	Cricket	Gumro	May- Sept	Wings discarded and fried with oil	
*Microcentum* sp. (?)	Orthoptera	Tettigoniidae	Katydid	Elbetto phiring	Aug- Oct	Roasted or fried with oil	
*Mantis* sp.	Mantodea		Preying mantis	Aasphiring	Aug- Oct	Fried with oil	
*Aristobia* sp.	Coleoptera	Cerambycidae	Long horn beetle	Chorgipok	June- Aug	Roasted or fried with oil	
*Batocera roylei* Hope 1833	Coleoptera	Cerambycidae	Long horn beetle	Chorgipok	June- Aug	Roasted or fried	
*Haplocerambyx* sp.	Coleoptera	Cerambycidae	Long horn beetle	Chorgipok	June- Sept	Roasted or fried with oil	
*Sternocera* sp.	Coleoptera	Buprestidae	Beetle	Keshkumari	June- Sept	Roasted or fried with oil	
*Helicupris* sp.	Coleoptera	Scarabaeidae	Beetle	Shimpok	June- Sept	Roasted or fried with oil	
Unidentified sp.	Coleoptera	Scarabaeidae	Beetle	Shimphoo	June- Sept	Roasted or fried with oil	
*Anomala* sp.	Coleoptera	Scarabaeidae	Beetle	Elphobang	Aug- Sept	Roasted or fried with oil	
*Propomacrus* sp.	Coleoptera	Scarabaeidae	Beetle	Phobang	June- Sept	Roasted or fried with oil	
*Lepidiota* sp.	Coleoptera	Scarabaeidae	Beetle	Phobang	Aug- Sept	Roasted or fried with oil	
*Lucanus laminifer* Waterhouse 1890.	Coleoptera	Lucanidae	Stag beetle	Komrengpok	June- Sept	Roasted or fried with oil	

The **Nocte** consume at least 16 species of insects, which include 4 Orthoptera, 5 Hymenoptera, 3 Hemiptera, 2 Isoptera and 1 species each belonging to the Odonata and Ephemeroptera (Table 2). Singpho people consume at least 14 species, which include 3 species each of the Orthoptera and Hemiptera; 4 species belong to the Hymenoptera and 2 each to the Isoptera and Coleoptera (Table 3).

**Figure 3:**
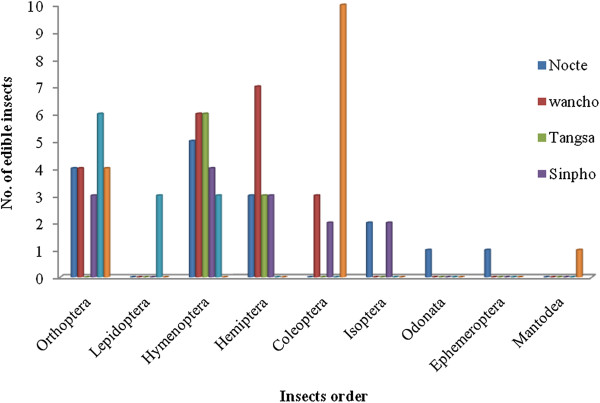
Comparative graphical representation of entomophagy of studied ethnic.

Members of the Tangsa (Mosang or Lumphi subtribes) comsume at least 9 species, of which 6 are Hymenoptera and 3 Hemiptera (Table. 4). Insects belonging to at least 12 species found acceptance as food by the Deori people. Six of the edible species were Orthoptera and 3 each were Lepidoptera and Hymenoptera (Table 5). The Chakma consume at least 15 species, which comprise 4 Orthoptera, 10 Coleoptera and 1 Mantodea (Table 6). Figure 3 represents the comparative graphical representation of entomophagy of the studied ethnic groups.

### Seasonal availability

Although insects generally occur throughout the year, their presence and abundance is determined by the presence and availability of their food plants as well as seasonal and local weather conditions. Reports on the seasonal abundance of the edible insects indicated that the maximum number of edible coleopteran species occurred between June and September (pre monsoon and monsoon); in winter and early spring, numbers of these insects dropped considerably. Acridid species occurred mostly during September and October, but gryllids were most abundant in the 4 months before, namely May through August.

Most of the bees and wasps (Hymenoptera) were consumed during the months November to February (winter season), but the weaver ant *Oecophylla smaragdina* was available throughout the year. Insects belonging to the order Hemiptera were reported to be restricted to the period of December to February, although at least one cicada and one pyrrhocorid bug (i.e., *Mictis tenebrosa)* were found to be available only between April to August (i.e., the pre-monsoon season). The hemipteran bug *Antilochus* sp*.* occurs year round according to the respondents.

Using insects to coincide with a particular season or period is one example of the richness of the traditional knowledge available in the local populations. Neupane *et al.*[12] investigated the rearing biology of *Samia ricini* in Nepal, where local people did not recommend rearing it during the cold months (November to April), because of the longer life cycle of 114–126 days during that time; they recommended instead the months of March to November, when life cycles were much shorter, amounting to just 38–61 days. Our study revealed that the Deori’s practice to rear this species during the months of April to September (mainly for the consumption of the larvae) had been passed down from generation to generation and agrees with the observation from Nepal. Sequestration of edible insects on a seasonal basis will undoubtedly have been an experience of the ethnic people of the area since ancient times and will have played an important role in the seasonally varied consumption of different insect species. However, a deeper study of this phenomenon had not been the objective of this initial survey. A detailed investigation of this aspect of entomophagy, although clearly worthwhile, would need to be carried out over at least a 12 month period by a researcher resident in the area or areas under debate.

### Stages and modes of insect consumption

Generally speaking, edible insects were consumed at all stages of development, i.e. egg, larva, pupa (when present), and adult. With Orthoptera and Hemiptera, adult stages were more highly valued than nymphs. Hymenoptera were eaten at all developmental stages, although eggs and honey were mentioned to be used particularly widely. It needs to be pointed out here that when the locals speak of ‘insect eggs’ they usually mean the pupal stage. For species belonging to the Vespidae, the larvae, i.e. the whitish grubs, were preferred. Pupae of the ant *Oecophylla smaragdina* were commonly consumed although some members of the Nocte tribe mentioned that they also ate the adults. Beetles, termites, mayflies, praying mantises were consumed as adults, whereas dragonflies were consumed exclusively in the aquatic larval stage. Likewise silk moths: they were consumed only when they were still caterpillars, ready to pupate or when they had just turned into a pupa.

The expressed preferences for different stages almost certainly depended on a variety of factors: palatability of the insects (which undoubtedly changes between developmental stages), availability and convenience with which the sought-after insects and their developmental stages can be obtained. Furthermore taboos or religious beliefs may be involved in the decision to avoid certain species and/or developmental stages [11].

### Comparisons between ethnic groups with respect to edible insects

Similarities and differences with regard to the entomophagic practices among the studied tribal groups exist. The total number of species, identified by us to be consumed by members of the Wangcho (Wancho) tribe, is 20. This, being the highest number of insect species used as food among the studied tribes, is followed by the Nocte, Chakma, Sinpho, Deori; the smallest number of insect species used as food, according to our survey, was 9 and associated with the Tangsa. Insect preferences and selectivity can be discussed in the context of species availability, but tribal tradition, culture and religious beliefs must also be considered, assuming that physiological differences of taste receptors between different human beings are negligible.

In the course of our survey it was noticed that of the other tribes in our study area, the Singpho were the most hesitant or shy to express that they habitually ate insects. However, kind persistence and expressing a positive attitude towards the consumption of insects by humans helped to create an environment of trust and made Singpho informants to reveal that they regularly consumed 14 different kinds of insects. Several people of the Singpho tribe expressed and accepted a negative attitude towards eating insects. Singpho tribals consume fewer orthopteran species than members of tribes that are mainly engaged in agricultural activities. Singpho people do not widely practice agriculture themselves, but get it done through other tribals, whom they engage to work in their fields. Those workers collect produce for the Singpho along with some of the Singpho’s preferred edible insects from the field. With respect to Orthoptera, Singpho consume only one acridid species and two species of gryllids. Singpho themselves are often engaged in tea plantation/cultivation and use yams and other edible tubers as their staple food.

Members of the Deori tribe practice mostly agriculture. They consume 6 orthopteran species (3 acridids, 1 tettigonid and 2 gryllids), which is the highest number among the studied tribes. Along with their agricultural activity, Deori tribals also practice weaving and culture three varieties of silk moth (Lepidoptera, Saturnidae), whose larvae and pupae (including those of a wild species) they consume. Except for the Deori, members of the other five tribes questioned by us, did not mention the intake of silk worm larvae at all, although a small number of respondents of the Singpho mentioned their inclination towards eating silkworm larvae.

The Tangsa (Mosang or Lumphi) were found not to consume Orthoptera unlike the other tribals mentioned above. If agricultural practices among the tribes influence their acceptance of at least a few orthopteran species, then this assumption does not hold true for the Tangsa, for they also practices shifting cultivation. Probably some other reason prompted them to discard or accept which species of insect they are willing to consume (e.g., they do take species of Hymenoptera and Hemiptera). Tangsa are followers of animistic beliefs, although they have also come under the influence of Theravada Buddhism [13,14] and this might have had some effect on their food insect preferences. This, however, is pure speculation and at the moment cannot be backed up with any evidence.

Members of the Wangcho (Wancho) tribe practice shifting cultivation, but their secondary activities include hunting and fishing for their livelihood. Besides using insects as food, they also use some species, mostly hemipterans, for therapeutic purposes. Out of 7 edible hemipteran species, 4 species were reported to be used only for therapeutic purposes, in particular for treating colds and coughs. A pharmacological assessment of the effectiveness of such treatment remains to be carried out. There are 6 hymenopteran species (occupying different ecological niches) and 3 species of beetle that were reported by our informants to be consumed by them. This demonstrates the dependence of these tribals on the forests and their products small and large, as they are still actively pursuing hunting activities. Four species of Orthoptera, including 2 gryllids and 2 acridids, were also taken as food by the Wangcho (Wancho).

Like the Wangcho (Wancho), the Nocte are also martial Naga tribals, but with their own distinctive life style, beliefs and social norms [15,16]. Remarkably, they mentioned termites, aquatic nymphs of dragonflies (Odonata), and mayflies (Ephemeroptera) as food insect items. The agricultural practice of the Nocte is primarily of the shifting type (jhum), although more and more people have begun to adopt terrace farming as well. In total 16 species, including 4 Orthoptera, 5 Hymenoptera, 3 Hemiptera, 2 Isoptera, 1 Odonata and 1 Ephemeroptera were readily accepted as food by the Nocte.

The Chakma, ethnically known to be Tibeto-Burman in origin and thus closely affiliated with tribes living at the foothills of the Himalaya, came to the state of Arunachal Pradesh as refugees. Most of them are employed in agriculture on a paid-up basis, usually working for the Singpho tribe in the same district. Our survey revealed that the Chakma consume the highest number of Coleopteran species amongst all of the studied tribes, i.e. 10 species of beetles, out of which 5 are Scarabaeidae, 3 Cerambycidae, 1 belongs to the Lucanidae and 1 to the Buprestidae. The insects are consumed both as grubs and as adults.

The acceptance of this high number of Coleopterans by the Chakma clearly indicates the dependence of these people on the forest’s insect inhabitants for their livelihood. Whatsoever of orthopteran species may be present in the field, they also take. Interestingly, our survey amongst the Chakma revealed that in contrast to the other tribes studied by us, no hymenopteran species were taken by them. Whether this is due to a conservation concept specifically aimed at bees, in which the by product of honey is valued more than the insect, or whether it has something to do with the ability of the bees to sting or bite and aggressively defend themselves can currently not be decided.

## Conclusion

What our survey has shown is that between different (and even neighbouring) tribes considerable differences can exist with regard to the numbers and species of insects used as food or used therapeutically. The Wangcho (Wancho) not only consume a wide variety of insects, they also reported the most extensive therapeutic uses of insects. However, in order to obtain reliable and comprehensive information on insect uses in folk medicinal practices, local healers not only need to be identified, they also have to be willing to share their knowledge with the investigator; and that is not always the case. The extensive information provided by the Wangcho (Wancho) with regard to the therapeutic uses of insects, therefore, could be a reflection of a less secretive society or a wider general awareness across the community of the usefulness of insects in health and healing when compared with other tribes. Alternatively, other tribes may simply not find much use for insects in treating sicknesses.

What our results cannot resolve is whether the differences in insect uses (be that for consumption or therapies) between the tribes have arisen out of the need to distinguish themselves from their neighbours or are primarily the consequence of the different environments and climatic conditions, which must affect the composition and abundance of insect species regionally. It is, for instance, obvious that most of the collections of Hemiptera and Hymenoptera take place during the months from November to March, while those of Orthoptera and Coleoptera species occur between May and September.

Agricultural practices between the different tribes can explain some (for example, the agricultural Deori consume mostly Orthopterans, but Singpho who do little work themselves in the fields, consider only one acridid worth eating), but not all differences (the Orthoptera-avoiding, agricultural Tangsa stand out as rather exceptional). Additional factors related to myths, taboos, etc. may therefore also contribute to what is deemed acceptable. It is clear, however, that such differences especially in those species taken as a regular food item (irrespective of what the differences are actually based on) lead to a more balanced and economic use of the food insect resource, since the pressure of exploiting this resource is distributed across a larger number of candidates.

## Competing interests

The authors declare that they have no competing interests.

## Authors’ contributions

JC carried out the field work and supervised SG’s research. SG participated in the field work and in the identification of the insects. VBM-R began the ethno entomological studies in North-East India and participated in the design, coordination, and preparation of the manuscript. All authors read and approved the final manuscript.

## References

[B1] Mitsuhashi J Sekai konchu shoko taizen 2008 Tokyo: Yasaka Shobo

[B2] Meyer-Rochow VB Chakravorty J Notes on entomophagy and entomotherapy generally and information on the situation in India in particular Appl Entomol Zool 2013 48 105 112 10.1007/s13355-013-0171-9

[B3] Meyer-Rochow VB Changkija S Uses of insects as human food in Papua New Guinea, Australia, and north-east India: cross-cultural considerations and cautious conclusions Ecol Food Nutr 1997 36 159 187 10.1080/03670244.1997.9991513

[B4] Meyer-Rochow VB Paoletti MP Traditional food insects and spider in several ethnic groups of north east India, Papua New Guinea, Australia and New Zealand Ecological implications of minilivestock: Rodents, frogs, snails, and insects for sustainable development 2004 Enfield, USA: Science Publ 385 409

[B5] Chakravorty J Ghosh S Meyer-Rochow VB Practices of entomophagy and entomotherapy by members of the Nyishi and Galo tribes of the state of Arunachal Pradesh (North-East India) J Ethnobiol Ethnomed 2011 7 5 10.1186/1746-4269-7-5 21235790 PMC3031207

[B6] Ghosh AK Sengupta T Handbook on insect collection, preservation and study 1982 Kolkata: Zool Surv India

[B7] Arrow GJ The fauna of India, including Pakistan, Ceylon, Burma and Malaya: Coleoptera Lamellicornia - Lucanidae and Passalidae. Volume IV 1949 New Delhi, India: Today and Tomorrows Printers and Publishers 1 274

[B8] Atkinman ET Fauna of Himalaya 1974 Delhi: Cosmos Publications

[B9] Vazirani TG Fauna of Coleoptera, family Gyrinidae and Haliplidae 1984 Kolkata: Zool Surv India 1 138

[B10] Gahan CJ The fauna of British India, including Ceylon and Burma: Coleoptera (Cerambycidae). Volume 1 1988 New Delhi, India: Today and Tomorrows Printers and Publishers 1 322

[B11] Meyer-Rochow VB Food taboos: their origins and purposes J Ethnobiol Ethnmed 2009 5 18 10.1186/1746-4269-5-18 PMC2711054 19563636

[B12] Neupane FP Thapa RB Parajulee MN Life and seasonal histories of the eri silkworm, *Samia Cynthia ricini* Hltt. (Lepidoptera: Saturniidae), in Chitwan, Nepal J Inst Agric Anim Sci 1990 11 113 120

[B13] Baveja JD New horizons of north east 1982 Nagpur, India: Western Book Depot 68

[B14] Roy S Rizvi SHM Tribal customary laws of north east India 1990 New Delhi: BR Publ Corp 34

[B15] Das NK Ethnic identity, ethnicity and social stratification in Northeast India 1989 New Delhi: Inter-India Publ

[B16] Fürer-Haimendorf C Return to the naked Nagas 1977 London: John Murray Publ

